# Diagnostic accuracy of MRI, CT, and [^18^F]FDG-PET-CT in detecting lymph node metastases in clinically early-stage cervical cancer — a nationwide Dutch cohort study

**DOI:** 10.1186/s13244-023-01589-1

**Published:** 2024-02-08

**Authors:** Ester P. Olthof, Brenda J. Bergink-Voorthuis, Hans H. B. Wenzel, Jordy Mongula, Jacobus van der Velden, Anje M. Spijkerboer, Judit A. Adam, Ruud L. M. Bekkers, Jogchum J. Beltman, Brigitte F. M. Slangen, Hans W. Nijman, Ramon G. V. Smolders, Nienke E. van Trommel, Petra L. M. Zusterzeel, Ronald P. Zweemer, Lukas J. A. Stalpers, Constantijne H. Mom, Maaike A. van der Aa

**Affiliations:** 1 Department of Research & Development, Netherlands Comprehensive Cancer Organisation (IKNL), Godebaldkwartier 419, DT Utrecht, 3511 The Netherlands; 2 Department of Gynecological Oncology, Amsterdam University Medical Center, Center for Gynecologic Oncology Amsterdam (CGOA), Amsterdam, The Netherlands; 3 Department of Health Technology and Services Research, Technical Medical Center, University of Twente, Enschede, The Netherlands; 4 Department of Obstetrics and Gynaecology, Zuyderland Medical Center, Heerlen, The Netherlands; 5 Department of Radiology, Amsterdam University Medical Center, Amsterdam, The Netherlands; 6 Department of Radiology and Nuclear Medicine, Amsterdam University Medical Centre, University of Amsterdam, Amsterdam, The Netherlands; 7 Department of Obstetrics and Gynaecology, Catharina Hospital, Eindhoven, The Netherlands; 8 Department of Obstetrics and Gynecology, Radboud University Medical Center, Nijmegen, The Netherlands; 9 Department of Obstetrics and Gynecology, Medical Center and GROW School for Oncology and Reproduction, Maastricht University, Maastricht, The Netherlands; 10 Department of Gynaecology, Leiden University Medical Center, Leiden, The Netherlands; 11 Department of Obstetrics and Gynaecology, University Medical Center Groningen, Groningen, The Netherlands; 12 Department of Gynaecological Oncology, Erasmus MC Cancer Institute University Medical Center, Rotterdam, The Netherlands; 13 Center for Gynaecologic Oncology Amsterdam (CGOA), The Netherlands Cancer Institute – Antoni van Leeuwenhoek, Amsterdam, The Netherlands; 14 Department of Gynaecological Oncology, University Medical Centre Utrecht, Utrecht Cancer Centre, Utrecht, The Netherlands; 15 Department of Radiation Oncology, Amsterdam University Medical Center, Amsterdam, The Netherlands

**Keywords:** Uterine cervical neoplasms, Lymphatic metastasis, Diagnostic imaging, Sensitivity and specificity

## Abstract

**Objectives:**

Imaging is increasingly used to assess lymph node involvement in clinically early-stage cervical cancer. This retrospective study aimed to evaluate the diagnostic accuracy of MRI, CT, and [^18^F]FDG-PET-CT.

**Methods:**

Women with International Federation of Gynaecology and Obstetrics (FIGO) 2009 stage IA2-IIA cervical cancer and pretreatment imaging between 2009 and 2017 were selected from the Netherlands Cancer Registry. Patient-based and region-based (i.e. pelvic and common iliac) nodal status was extracted from radiology reports. Pathology results were considered the reference standard for calculating accuracy indices. Multiple imputation was used for missing pathology to limit verification bias risk.

**Results:**

Nodal assessment was performed in 1676 patients with MRI, 926 with CT, and 379 with [^18^F]FDG-PET-CT, with suspicious nodes detected in 17%, 16%, and 48%, respectively. [^18^F]FDG-PET-CT was used to confirm MRI/CT results in 95% of patients. Pathology results were imputed for 30% of patients. [^18^F]FDG-PET-CT outperformed MRI and CT in detecting patient-based nodal metastases with sensitivities of 80%, 48%, and 40%, and AUCs of 0.814, 0.706, and 0.667, respectively, but not in specificity: 79%, 92%, and 92%. Region-based analyses showed similar indices in the pelvic region, but worse performance in the common iliac region with AUCs of 0.575, 0.554, and 0.517, respectively.

**Conclusions:**

[^18^F]FDG-PET-CT outperformed MRI and CT in detecting nodal metastases, which may be related to its use as a verification modality. However, MRI and CT had the highest specificity. As MRI is generally performed routinely to assess local and regional spread of cervical cancer, [^18^F]FDG-PET-CT can be used to confirm suspicious nodes.

**Critical relevance statement:**

Accurate assessment of the nodal status in clinically early-stage cervical cancer is essential for tumour staging, treatment decision making and prognosis.

**Key points:**

• The accuracy of MRI, CT or [^18^F]FDG-PET-CT for nodal staging in early cervical cancer is a subject of discussion.

• Overall, [^18^F]FDG-PET-CT outperformed MRI, followed by CT, when used as a verification modality.

• Staging with MRI and the addition of [^18^F]FDG-PET-CT to verify high-risk cases seems to be a good approach.

**Graphical Abstract:**

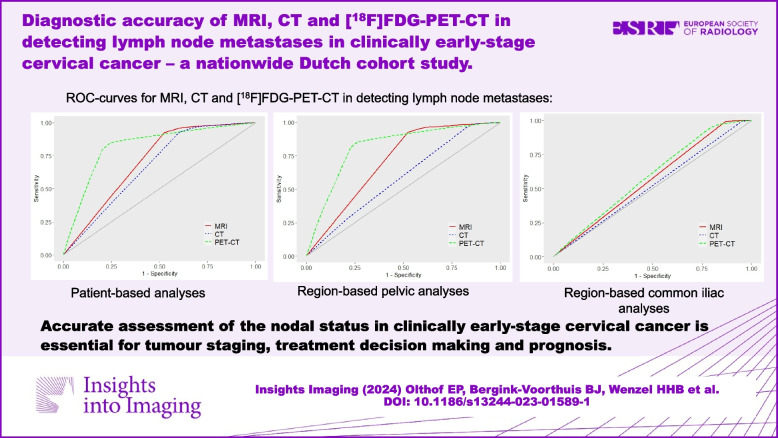

**Supplementary Information:**

The online version contains supplementary material available at 10.1186/s13244-023-01589-1.

## Introduction

Cervical cancer is the fourth most common cancer in women worldwide, representing 604,000 new cases and 342,000 deaths in 2020 [1]. One of the most important prognostic factors in cervical cancer is lymph node involvement, a factor included in the revised International Federation of Gynaecology and Obstetrics (FIGO) system in 2018. In this FIGO system, pelvic and para-aortic lymph nodes suspicious for metastasis on imaging are classified as stage IIIC1 and IIIC2, respectively, with the annotation ‘r’ (radiologic), indicating that the role of imaging in the staging and management of cervical cancer has increased [2, 3].

Accurate assessment of the nodal status is essential when deciding on treatment options. In early-stage cervical cancer, the nodal status determines whether radical hysterectomy or (chemo)radiotherapy is recommended [4]. In (chemo)radiotherapy, suspicious nodes on imaging may influence radiotherapy settings (i.e. extended-field and nodal boosting). Imaging-based treatment modifications are observed in approximately 13% of patients with early-stage cervical cancer, with MRI, CT or [^18^F]FDG-PET-CT being the most commonly used modalities [4, 5]. The current Dutch guidelines recommend the use of MRI for clinical staging of patients with early-stage cervical cancer, because of its accuracy in determining tumour size and local spread, while [^18^F]FDG-PET-CT is recommended as a verification modality for the validation of suspicious nodes [6, 7] However, due to the lack of consensus, the use of imaging modalities in clinical practice remains variable.

The performance of these techniques has been described in several meta-analyses, reporting an overall pooled sensitivity and specificity of 41–57% and 93–98% for MRI, 51–59% and 87–92% for CT, and 52–78% and 92–95% for [^18^F]FDG-PET-CT [8–10]. However, these results are mainly based on outdated retrospective data, with a high risk of selection bias, as pathological verification of suspicious nodes on imaging is often partially lacking, as patients with suspicious nodes usually receive primary chemoradiotherapy. This form of selection bias, where the reference standard (i.e. pathological examination of lymph nodes) is not performed in all patients, is also known as partial verification bias and can lead to biased accuracy estimates [11]. Therefore, the accuracy of nodal imaging by MRI, CT, and [^18^F]FDG-PET-CT is still controversial and their performance may have improved over time due to technological advances.

As imaging is increasingly used for nodal staging in cervical cancer patients, we believe it is necessary to provide diagnostic indices of pretreatment imaging based on a more recent and larger cohort of patients, while taking into account the risk of partial verification bias. Therefore, the present study aimed to evaluate the diagnostic accuracy of MRI, CT, and [^18^F]FDG-PET-CT for lymph node metastases in clinically early-stage cervical cancer, on a patient-based and region-based (i.e. pelvic and common iliac) level.

## Methods

### Study design

We performed a nationwide, retrospective, cohort study by analysing data between 2009 and 2017 from the Netherlands Cancer Registry, after Privacy Review Board approval (No K22.262). This registry holds population-based data containing > 95% of all cancer patients in the Netherlands since 1989. Patients with FIGO (2009) stage IA2-IIA cervical cancer and pretreatment nodal status assessment by MRI, CT, and/or [^18^F]FDG-PET-CT, were eligible for this study. Patients were excluded if pathological examination of lymph nodes was obtained > 8 weeks after imaging, as prolonged intervals might increase the risk of inaccuracy.

Trained data managers collected additional data on lymph node metastases from hospital records. Lymph node status was recorded for five nodal regions (i.e. pelvic left/right, common iliac left/right and para-aortic) as suspicious, inconclusive, negative or unknown, as reported by the radiologist. Per patient, the nodal status of all regions was combined for patient-based analyses, and the laterality was combined for region-based analyses, according to the order mentioned above. Inconclusive nodes were first considered suspicious and later negative in subgroup analyses to explore the robustness of our findings and to assess how different interpretations of inconclusive results may affect the diagnostic accuracy. If reported, the short-axis diameter was recorded for positive or inconclusive nodes. Although there are no (inter)national protocols available, lymph nodes in cervical cancer are generally considered suspicious when they have a short axis diameter ≥ 1.0 cm, morphological tumour features (i.e. central necrosis) and/or increased FDG uptake (more than the adjacent vessel) [12, 13]. All MRI, CT, and [^18^F]FDG-PET-CT scans were performed according to local protocols, with [^18^F]FDG-PET-CT scans following the Dutch (Nedpas) and international (EARL) standards [14]. As most patients (94%) were referred to specialised oncology centres, it is likely that the majority of scans were interpreted by experienced radiologists and nuclear medicine physicians.

Pathological examination of the lymph nodes was considered the reference standard. Examination could be performed by lymphadenectomy, debulking surgery, sentinel lymph node biopsy or fine-needle cytology or biopsy. The pathological lymph node status was also recorded for the five nodal regions. The sentinel lymph nodes’ laterality, but not the region, was registered, though considered to be pelvic as this is the case in > 93% of sentinel nodes in cervical cancer [15]. According to current guidelines, isolated tumour cells (≤ 0.2 mm) on pathological examination were not considered to be lymph node metastases [4]. Pathological nodal status was considered missing if patients were treated with neoadjuvant chemotherapy prior to pathological examination. Furthermore, data on patient and tumour characteristics were also collected. Direct conversion to FIGO 2018 was not possible due to missing information on horizontal spread.

### Statistical analysis

Multiple imputation has been described as a reliable method to reduce partial verification bias, even when data are not missing at random, as in our case [11]. Therefore, we imputed the pathological nodal status when missing, using multivariate imputation by chained equations (MICE) with 20 imputations (Supplementary Table 1–3) [16]. We repeated this procedure twice, for the patient- and region-based analyses, and established the validity by reviewing convergence plots and comparing original and imputed data. We applied Rubin’s rule to combine the sensitivity, specificity, positive predictive value (PPV), negative predictive value (NPV), and area under the receiver operating curve (AUC) of all imaging modalities for detecting lymph node metastases in the imputed data [17, 18].

The para-aortic region was excluded from region-based analyses because para-aortic lymphadenectomies are not routinely performed in the Netherlands, resulting in too few patients with pathological verification. Subgroup analyses included patient cohorts with > 1 imaging modality and recalculation of diagnostic indices after considering an inconclusive nodal status as negative. The Wilcoxon signed-rank test was used to compare paired data without a normal distribution. Confidence intervals for AUCs were calculated using the DeLong test and compared using the chi-squared test; *p*-values below 0.05 were considered statistically significant. South Texas Art Therapy Association SE 17 (StataCorp, College Station, TX, USA) and R software were used for all analyses.

## Results

### Baseline characteristics

In total, 2236 patients with early-stage cervical cancer were included (Supplementary Fig. 1), whose baseline characteristics are presented in Table 1. Nodal evaluation was performed in 1676 (75%) patients by MRI, and in 926 (41%) and 379 (17%) patients by CT and [^18^F]FDG-PET-CT, respectively. The rate of MRI and [^18^F]FDG-PET-CT imaging increased over time from 7–8% to 16%, while the rate of CT decreased from 14 to 9%. Suspicious nodes were observed in 286 (17%) patients on MRI, 148 (16%) on CT, and 183 (48%) on [^18^F]FDG-PET-CT. The rate of suspicious nodes remained constant over the years, within a range of 15–21% (*p* = 0.56). Of all patients, suspicious nodes on MRI, CT, or [^18^F]FDG-PET-CT were located in the pelvic, common iliac and para-aortic regions in 18% (*n* = 393), 2% (*n* = 54), and 3% (*n* = 70), respectively. The median short-axis of these nodes was 11 mm (range 5–50) in the pelvic region, 9 mm (range 6–29) in the common iliac region (*p* = 0.013) and 10 mm (5–28) in the para-aortic region. In 361/379 (95%) patients who underwent [^18^F]FDG-PET-CT, MRI and/or separate CT were also performed. Neoadjuvant therapy was administered to 89 patients (4%). Pathologic assessment of the nodal status was available in 1557 (70%) patients, mainly by lymphadenectomy (97%; *n* = 1517), with a prevalence of nodal metastases of 19% (*n* = 234), 24% (*n* = 142) and 44% (*n* = 60) in the MRI, CT, and [^18^F]FDG-PET-CT groups, respectively, which increased to 24% (*n* = 402), 26% (*n* = 241) and 46% (*n* = 174) after imputation.

**Table 1:** Baseline characteristics

Characteristics	*n* = 2236	
	*n*/median	%/range
Age, years	44	19–102
BMI, kg/m^2^	25	15–77
FIGO 2009 stage		
IA2	57	2.6
IB1	1554	69.5
IB2	349	15.6
IIA1	158	7.1
IIA2	118	5.3
Tumour size, mm	30	0–150
Histology		
Squamous cell carcinoma	1487	66.5
Adenocarcinoma	602	26.9
Adenosquamous cell carcinoma	100	4.5
Neuroendocrine carcinoma	35	1.6
Other	12	0.5
Type of imaging		
MRI	1676	75.0
CT	926	41.4
[^18^F]FDG-PET-CT	379	17.0
MRI and CT	384	17.2
MRI and [^18^F]FDG-PET-CT	314	14.0
CT and [^18^F]FDG-PET-CT	106	4.7
MRI, CT, and [^18^F]FDG-PET-CT	59	2.5
Short-axis of suspicious pelvic node, mm^a^	10	5–50
Short-axis of suspicious common iliac node, mm^a^	9	6–29
Patient-based nodal status on MRI		
Negative	1390	82.9
Inconclusive	89	5.3
Positive	197	11.8
Patient-based nodal status on CT		
Negative	778	84.0
Inconclusive	53	5.7
Positive	95	10.3
Patient-based nodal status on [^18^F]FDG-PET-CT		
Negative	196	51.7
Inconclusive	21	5.5
Positive	162	42.7
Region with positive nodal status on imaging^b^		
Pelvic	393	17.7
Common iliac	54	2.4
Para-aortic	70	3.1
Patient-based nodal status on pathology		
Negative	1240	55.5
Positive	317	14.2
Unknown	679	30.4
Time between imaging and pathological examination, days		
MRI	25	1–56
CT	26	1–56
[^18^F]FDG-PET-CT	20	0–44
Nodal examination		
Absent	679	30.4
Lymphadenectomy	1517	67.8
Nodal debulking	32	1.4
Biopsy/fine-needle aspiration	2	0.1
Intraoperative frozen section	4	0.2
Sentinel node biopsy only	2	0.1

*Abbreviations: n *number of patients, *BMI *body mass index, *FIGO *International Federation of Gynaecology and Obstetrics

^a^For positive and inconclusive nodes only

^b^Including a positive and inconclusive nodal status at MRI, CT, or [^18^F]FDG-PET-CT

### Patient-based diagnostic accuracy

**Fig. 1:**
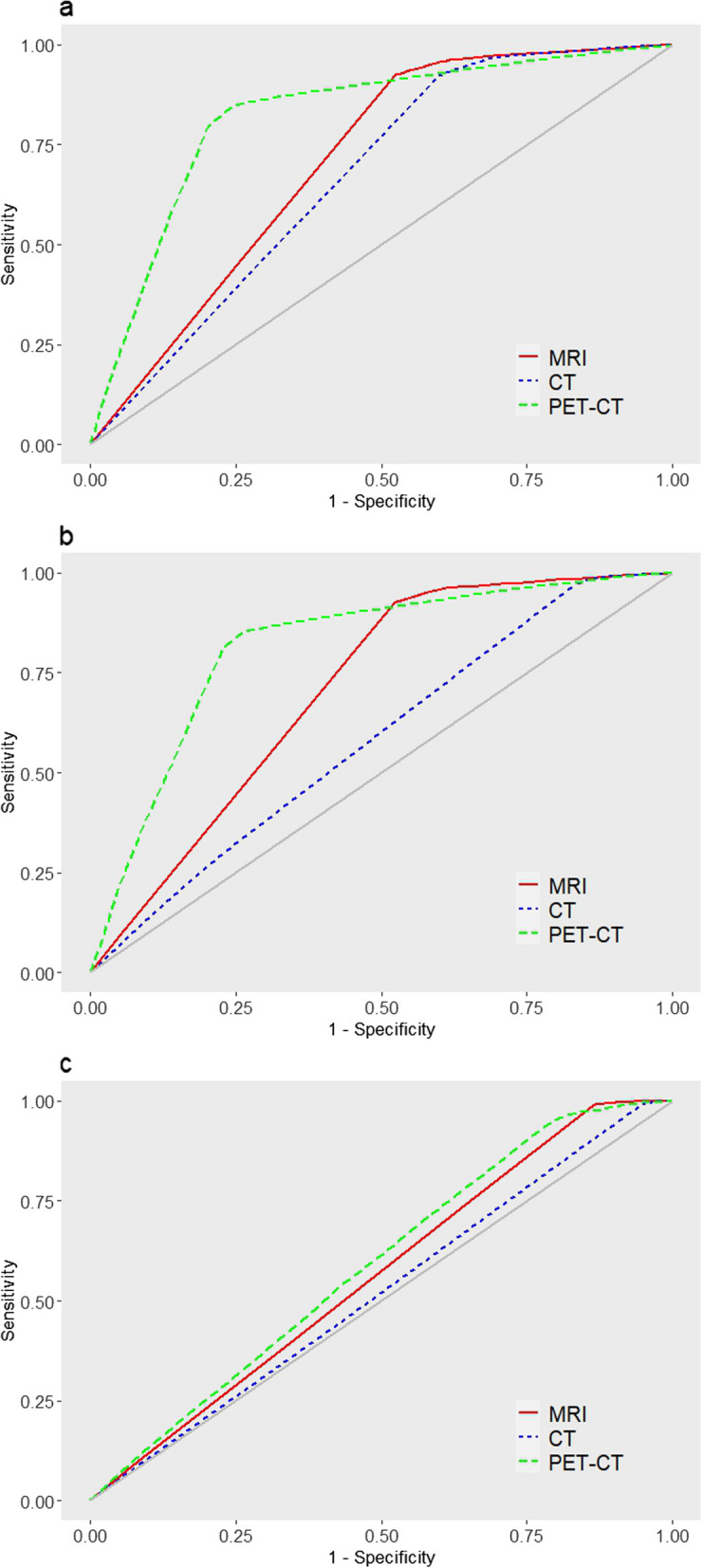
ROC-curves for MRI, CT, and [^18^F]FDG-PET-CT in detecting lymph node metastases regarding (**a**) patient-based, (**b**) region-based pelvic, and (**c**) region-based common iliac analyses

The accuracy of MRI, CT, and [^18^F]FDG-PET-CT in detecting lymph node metastases on a patient-based level of original and imputed data are shown in Table 2. [^18^F]FDG-PET-CT outperformed MRI and CT in sensitivity (80% vs 48% and 40%, respectively), but not in specificity (79% vs 92% and 92%, respectively), resulting in an AUC of 0.814 vs 0.706 and 0.667 (*p* = 0.003, imputed data), as shown in Fig. 1a. [^18^F]FDG-PET-CT had the highest PPV (76%), while MRI had the highest NPV (85%). All indices increased or remained stable after imputation, as did the prevalence of lymph node metastases (from 19–44% to 24–46%).

**Table 2:** Patient-based diagnostic indices for MRI, CT, and [^18^F]FDG-PET-CT in detecting lymph node metastases based on original and imputed data

Modality	Prev LNM	Sensitivity	Specificity	PPV	NPV	AUC^a^
Original data						
MRI	19 (17–22)	34 (31–36)	93 (92–94)	54 (52–57)	85 (83–87)	0.639 (0.607–0.670)
CT	24 (21–28)	37 (33–41)	91 (89–93)	57 (53–61)	82 (79–85)	0.646 (0.603–0.688)
[^18^F]FDG-PET-CT	44 (35–52)	73 (66–81)	77 (70–84)	71 (63–79)	79 (72–86)	0.787 (0.714–0.860)
Imputed data						
MRI	24 (22–26)	48 (45–50)	92 (91–94)	66 (64–69)	85 (83–87)	0.706 (0.674–0.737)
CT	26 (23–29)	40 (37–43)	92 (91–94)	64 (61–67)	82 (79–84)	0.667 (0.630–0.704)
[^18^F]FDG-PET-CT	46 (41–51)	80 (76–84)	79 (75–83)	76 (72–81)	82 (78–86)	0.814 (0.752–0.876)

Numbers represent % with (95% confidence interval)

*Abbreviation:* *Prev LNM *prevalence of lymph node metastases

^a^AUC without dichotomising the nodal status on imaging

Subgroup analyses of patient cohorts with > 1 imaging modality after imputation included samples ranging from 59 to 384 patients, depending on the combination of MRI, CT, and/or [^18^F]FDG-[^18^F]FDG-PET-CT (Supplementary Table 4). Within these cohorts, the AUCs of all three modalities after imputation were nearly equivalent to those in the original patient-based analyses (± 0.005–0.072). As in the original analyses, the AUC of [^18^F]FDG-PET-CT was consistently higher than of MRI and CT in all cohorts, although not significantly (*p* = 0.58 imputed data), while the AUC of MRI was generally higher than CT. Nodal status discordance between one of the three imaging modalities was observed in 20/59 (34%) patients.

The prevalence of metastatic nodes after imputation was determined for cohorts with different combinations of MRI, CT, and/or [^18^F]FDG-PET-CT results (Supplementary Table 5). The prevalence of nodal metastases in cohorts with discrepancy in the nodal status between two imaging modalities (14–73%) was substantially higher compared to the total cohort with a negative MRI, CT or [^18^F]FDG-PET-CT (15–18%), especially in the case of a positive [^18^F]FDG-PET-CT (58–73%).

### Region-based diagnostic accuracy

Table 3 shows the performance of MRI, CT, and [^18^F]FDG-PET-CT in detecting lymph node metastases on a region-based level for original and imputed data. The prevalence of nodal metastases and the accuracy of the different diagnostic modalities in the pelvic region were highly comparable to the patient-based results. [^18^F]FDG-PET-CT outperformed MRI and CT with respect to the AUC (0.803 vs 0.705 and 0.656; Fig. 1b), the sensitivity (77% vs 47% and 37%), and PPV (76% vs. 66% and 64%), respectively. In contrast, inferior performance was observed for specificity (80% vs. 93% and 93%) and NPV (81% vs. 85% and 81%, respectively).

**Table 3:** Region-based diagnostic indices for MRI, CT, and [^18^F]FDG-PET-CT in detecting lymph node metastases based on original and imputed data

Modality	Region	Prev. LNM	Sensitivity	Specificity	PPV	NPV	AUC^a^
Original data							
MRI	Pelvic	19 (16–21)	33 (30–36)	93 (92–95)	53 (50–56)	86 (84–88)	0.631 (0.599–0.663)
	Common iliac	4 (3–5)	10 (8–12)	99 (99–100)	44 (41–48)	96 (95–98)	0.549 (0.500–0.597)
CT	Pelvic	23 (20–27)	32 (28–36)	92 (90–94)	54 (50–58)	82 (79–85)	0.620 (0.578–0.661)
	Common iliac	4 (2–6)	0 (–)	99 (99–100)	0 (–)	96 (94–98)	0.497 (0.493–0.500)
[^18^F]FDG-PET-CT	Pelvic	42 (34–51)	70 (62–77)	80 (73–87)	80 (73–87)	72 (65–80)	0.750 (0.674–0.825)
	Common iliac	16 (9–23)	19 (11–26)	98 (95–100)	60 (50–69)	87 (80–93)	0.582 (0.482–0.682)
Imputed data							
MRI	Pelvic	23 (21–25)	47 (45–50)	93 (91–94)	66 (63–68)	85 (84–87)	0.705 (0.675–0.736)
	Common iliac	8 (7–9)	12 (10–13)	99 (99–100)	56 (53–58)	93 (92–94)	0.554 (0.508–0.600)
CT	Pelvic	25 (22–28)	37 (34–41)	93 (91–95)	64 (61–67)	81 (79–84)	0.656 (0.615–0.697)
	Common iliac	8 (7–10)	4 (3–5)	99 (99–100)	33 (30–36)	92 (90–94)	0.517 (0.487–0.547)
[^18^F]FDG-PET-CT	Pelvic	45 (40–50)	77 (73–82)	80 (76–84)	76 (72–80)	81 (77–85)	0.803 (0.725–0.881)
	Common iliac	22 (18–26)	20 (16–24)	95 (92–97)	51 (46–56)	81 (77–85)	0.575 (0.489–0.661)

Numbers represent % with (95% confidence interval)

*Abbreviation: Prev LNM* prevalence of lymph node metastases

^a^AUC without dichotomising the nodal status on imaging

Comparing the performance of MRI, CT, and [^18^F]FDG-PET-CT in the common iliac region with the pelvic region, the AUCs (0.554, 0.517, and 0.575; Fig. 1c), sensitivities (12%, 4%, and 20%), and PPVs (56%, 33%, and 51%), respectively, were considerably lower in the common iliac region. On the other hand, this region had equivalent or higher specificities (99%, 99%, and 95%) and NPVs (93%, 92%, and 81%) for MRI, CT, and [^18^F]FDG-PET-CT, respectively. Again, [^18^F]FDG-PET-CT outperformed MRI and CT in terms of AUC and sensitivity. The prevalence of common iliac metastases (8–22%) was substantially lower than that of pelvic metastases (23–45%) for all modalities.

### Inconclusive lymph nodes regarded as negative

Patient- and region-based diagnostic indices were recalculated and changed minimally after inconclusive lymph nodes (5–6%) were considered negative instead of suspicious (Supplementary Table 6 and 7). The sensitivity of MRI, CT, and [^18^F]FDG-PET-CT in detecting nodal metastases on a patient-based level decreased to 38%, 31% and 75%, the NPV to 83%, 80%, and 80%, and the AUC to 0.671, 0.636, and 0.795, respectively. Conversely, the specificity (96%, 97%, and 85%) and PPV (77%, 77%, and 81%) of all three modalities increased after inconclusive statuses were included as negative. Similar trends were observed in the pelvic and para-aortic regions.

## Discussion

In this study, we evaluated the diagnostic performance of pretreatment imaging for lymph node metastases in recent years in clinically early-stage cervical cancer on a patient- and region-based level, while reducing the risk of partial verification bias by multiple imputation. [^18^F]FDG-PET-CT was superior in detecting nodal metastases (sensitivity/PPV) at both levels, compared to MRI and CT. Although, this is probably related to its use as a verification modality. In contrast, MRI and CT had the highest specificity. The accuracy of all three modalities was lower in the common iliac than the pelvic region, especially regarding sensitivity. In addition, there may be a significant risk of nodal involvement in the case of multiple imaging with at least one positive result, particularly a positive [^18^F]FDG-PET-CT. Based on our results, we believe that verification with [^18^F]FDG-PET-CT may be valuable in differentiating between patients at low and high risk of metastasis, particularly in cases of suspicious nodes on MRI. However, caution should be exercised when using this information to guide treatment planning because of the risk of false-positive or false-negative results, especially for FIGO 2018 stage IIIC ‘r’ involving the common iliac region.

Consistent with our findings, previous studies have demonstrated that [^18^F]FDG-PET-CT has an overall higher diagnostic performance than MRI and CT in detecting nodal metastases in patients with cervical cancer [9, 10, 19, 20]. The outperformance of [^18^F]FDG-PET-CT can be explained by the following. Advantages of functional imaging: [^18^F]FDG-PET-CT detects potential metastases due to increased glucose metabolism, whereas MRI and CT rely mainly on nodal size (≥ 1 cm) and morphology [21]. In addition, [^18^F]FDG-PET-CT imaging fields generally cover a more comprehensive area than MRI and CT. Therefore, more lymph node metastases can be detected, including those outside the pelvis. However, the higher accuracy of [^18^F]FDG-PET-CT in our study may also be explained by its use as a verification modality, as 95% of our patients with [^18^F]FDG-PET-CT had an MRI or CT previously. Previous MRI and/or CT findings may have influenced the interpretation of the [^18^F]FDG-[^18^F]FDG-PET-CT scan by the nuclear medicine physician. In addition, the prevalence of lymph node metastases in the [^18^F]FDG-PET-CT group was nearly twice the prevalence with MRI and CT. As [^18^F]FDG-PET-CT is recommended by the Dutch guidelines for the validation of suspicious nodes, patients receiving [^18^F]FDG-PET-CT will have a higher probability of suspicious nodes and nodal metastases, as reflected in our study. Verification of MRI/CT results with [^18^F]FDG-PET-CT seems useful to identify patients at high risk of metastasis, particularly in cases with suspicious nodes on MRI. Our results suggest that this strategy reduces the risk of unwarranted omission of surgery or, in case of primary chemoradiotherapy, overtreatment with nodal boosting/extended-field (fewer false-positives). However, due to the low sensitivity of MRI (more false negatives), patients may require adjuvant chemoradiotherapy due to postoperative pathological detection of lymph node metastases missed by pretreatment imaging. And in the case of primary radiotherapy, the low sensitivity of MRI may result in undertreatment because of inadequate radiotherapy settings.

In the region-based analyses, all three modalities showed higher accuracy in the pelvic region than in the common iliac region, especially in terms of sensitivity. Cervical cancer generally metastasises via the lymphatic system, where the common iliac region is considered a secondary lymphatic drainage station [22, 23]. The size of metastatic lymph nodes in this region may be smaller. Therefore, metastases may be harder to detect, as demonstrated in our study. These findings align with the literature where higher sensitivities have been demonstrated in the pelvic region than in the para-aortic region [8, 24–26]. For MRI, the resolution setting is generally lower for the common iliac than the pelvic region, which may have contributed to its lower accuracy in this region. Nevertheless, the identification of metastatic nodes in secondary stations is important, because they are associated with a poor prognosis and, as a consequence, extended field radiotherapy is often recommended [4, 27]. According to our results, metastatic nodes in the common iliac region are underdiagnosed. Therefore, patients are at risk of undertreatment when receiving primary chemoradiotherapy, due to inadequate radiotherapy-field settings. Meanwhile, patients are at risk of receiving adjuvant therapy after surgery due to lymph node metastases.

In the literature, the diagnostic accuracy of MRI, CT, and [^18^F]FDG-PET-CT varies in detecting lymph node metastases in cervical cancer, possibly related to different study designs, definitions of suspicious nodes, imaging techniques, and heterogeneous patient cohorts. Sensitivities and specificities were reported to be 24–73% and 69–96% for MRI, 33–67% and 56–97% for CT, and 35–91% and 90–100% for [^18^F]FDG-PET-CT. The PPV and NPV were 48–67% and 78–98% for MRI, 20–86% and 72–93% for CT, and 47–100% and 81–96% for [^18^F]FDG-PET-CT. Corresponding metastatic nodal prevalence rates were 16% to 34% [8–10, 20, 28–35]. Most of our rates fall within the broad ranges described in the literature, although we found a slightly lower specificity and a higher metastatic rate for [^18^F]FDG-PET-CT. As mentioned before, this may be related to the use of [^18^F]FDG-PET-CT as a verification modality in our cohort. In addition, all metastatic rates increased after imputation, which was expected, as pathological verification is often lacking in patients with poor prognostic factors who are at risk of metastasis (e.g. suspicious nodes and larger tumour size). Consequently, these patients are often excluded from both prospective and retrospective studies, leading to biased estimates of diagnostic indices.

By means of a retrospective study design, we provided the diagnostic indices of three imaging modalities within one large, nationwide cohort. However, there are several limitations. We used multiple imputation to account for partial verification bias. Although the imputation rates were high (30–40%), the variable distributions after imputation were similar to the original data after imputation, except for the prevalence of pathological nodal metastases, which was expected and explained above. Other potential factors influencing our results include intra- and inter-observer variability, as nodal status was recorded in different centres over an extended period of time (2009 to 2017). Differences in imaging techniques may have introduced variability into our results, but adjustment for these technical variations was unfortunately not possible, as detailed data on the technical parameters are not available. On the other hand, our results provide insight into the diagnostic performance in the daily Dutch clinical practice. Finally, our results are mostly based on conventional imaging techniques, as our data cover the years 2009–2017. For future studies, it would be interesting to include more advanced techniques such as diffusion-weighted (DW)-MRI, which may increase the sensitivity to 86–87% and reduce the need for verification by [^18^F]FDG-PET-CT [9, 36].

In conclusion, [^18^F]FDG-PET-CT outperformed MRI and CT in detecting nodal metastases in patients with early-stage cervical cancer with a sensitivity of 80%, when used as verification modality, while MRI and CT had the highest specificity (92%). In other words, MRI might be the preferred imaging modality for pretreatment staging cervical cancer patients by accurately excluding patients without nodal metastases, next to determining tumour size and local spread. [^18^F]FDG-PET-CT may be added in patients with suspicious nodes on MRI or in patients at high risk of nodal metastases (e.g. large tumour size and increased tumour marker). However, this hypothesis should be confirmed in prospective studies before clinical implementation. Finally, accounting for partial verification bias increased almost all diagnostic indices, suggesting that diagnostic performance in previous studies based on retrospective data may have been underestimated.

### Supplementary Information

**Additional file 1: Supplementary Table 1. **Imputation models.** Supplementary Table 2.** Distribution of variables with missing data before and after multiple imputation on patient-based level. **Supplementary Table 3.** Distribution of variables with missing data before and after multiple imputation on region-based level. **Supplementary Table 4.** Patient-based diagnostic indices for MRI, CT and [18F]FDG-PET-CT in detecting lymph node metastases of patient cohorts with multiple imaging results. **Supplementary Table 5.** The prevalence of lymph node metastases in patient cohorts according to (multiple) imaging results. **Supplementary Table 6.** Patient-based diagnostic indices for MRI, CT and [18F]FDG-PET-CT in detecting lymph node metastases based on original and imputed data, inconclusive nodes considered negative. **Supplementary Table 7.** Region-based diagnostic indices for MRI, CT and [18F]FDG-PET-CT in detecting lymph node metastases based on original and imputed data, inconclusive nodes considered negative. **Supplementary Figure S1.** Patient-flow chart of patient-based analyses according to Standards for Reporting of Diagnostic Accuracy (STARD).

## Data Availability

The data that support the findings of this study are available from the corresponding author upon reasonable request.

## Abbreviations

[^18^F]FDG: 2-Deoxy-2-[fluorine-18]fluoro-D-glucose
AUC: Area under the receiver operating curve
FIGO: International Federation of Gynaecology and Obstetrics
NPV: Negative predictive value
PPV: Positive predictive value
‘r’: Radiologic
