# Supplementary material for: Diagnostic accuracy of MRI, CT, and [18F]FDG-PET-CT in detecting lymph node metastases in clinically early-stage cervical cancer — a nationwide Dutch cohort study
Source: Insights Imaging. 2024 Feb 8;15:36. doi: 10.1186/s13244-023-01589-1 (PMC10853153; doi:10.1186/s13244-023-01589-1)

Diagnostic accuracy of MRI, CT and [^{18}F]FDG-PET-CT in detecting lymph node metastases in clinically early-stage cervical cancer – a nationwide Dutch cohort study

ELECTRONIC SUPPLEMENTARY MATERIAL

Supplementary Table 1

Imputed model	Included variables
Patient-based	Pathologic nodal status, clinical nodal metastasis status according to TNM 8, age, lympho-vascular space invasion, FIGO 2009 stage, primary tumour size, grade, histology, depth of invasion, horizontal spread, suspicion of parametrial invasion, nodal status and short-axis at MRI, CT, [^{18}F]FDG-PET-CT
Region-based	Pathologic nodal status of the pelvic and common iliac regions, clinical nodal metastasis status according to TNM 8, age, lympho-vascular space invasion, FIGO 2009 stage, primary tumour size, grade, histology, depth of invasion, horizontal spread, suspicion of parametrial invasion, pelvic and common iliac nodal status and short-axis at MRI, CT, [^{18}F]FDG-PET-CT

Abbreviations: FIGO, International Federation of Gynaecology and Obstetrics.

Supplementary Table 2. Distribution of variables with missing data before and after multiple imputation on patient-based level.

Imputed variables	Missing	Original data	Imputed data
Pathologic nodal metastasis	679 (30.4%)		
Positive		20.4	23.6
Negative		79.6	76.4
Tumour grade	543 (29.9%)		
1		11.6	11.7
2		47.2	47.0
3		40.8	40.8
4		0.5	0.5
cN	114 (5.1%)		
0		83.9	83.9
1		16.1	16.1
Tumour grade	720 (32.2%)		
1		11.5	11.7
2		47.2	47.2
3		40.8	40.6
4		0.5	0.5
LVSI	492 (22.0%)		
Present		42.4	41.7
Absent		57.6	58.3
Depth of invasion, mm	729 (32.6%)		
<3		16.9	14.5
3-5		22.4	20.6
>5		60.7	65.0
Nodal short-axis category, mm [a]	56 (2.5%)		
Not suspicious		83.5	81.4
<10		8.2	9.4
10-19		6.5	7.2
≥20		1.9	2.0
Suspicion of parametrial invasion	392 (17.5%)		
Absent		92.6	91.6
Presumably absent		1.7	1.9
Presumably present		5.7	6.5
Tumour size, cm	49 (2.2)		
≤2		31.0	30.6
<4		8.2	8.2
>2-4		33.4	33.3
>4		27.4	27.9

Numbers represent % or number of patients. [a] negative nodes were allocated as category '0'.

Abbreviations: cN, clinical nodal status; LVSI, lympho-vascular space invasion.

Supplementary Table 3. Distribution of variables with missing data before and after multiple imputation on region-based level.

Imputed variables	Missing	Original data	Imputed data
Pathologic nodal metastasis			
Pelvic	691 (30.9%)		
Positive		19.5	22.8
Negative		80.5	77.2
Common iliac	921 (41.2%)		
Positive		4.0	7.8
Negative		96.0	92.2
Tumour grade	720 (32.2%)		
1		11.5	11.6
2		47.2	47.1
3		40.8	40.7
4		0.5	0.6
cN	114 (5.1%)		
0		83.9	83.9
1		16.1	16.1
LVSI	492 (22.0%)		
Present		42.4	41.4
Absent		57.6	58.6
Depth of invasion, mm	729 (32.6%)		
<3		16.9	14.6
3-5		22.4	20.5
>5		60.7	64.9
Short-axis of suspicious pelvic node, mm [a]	32 (1.4%)		
Not suspicious		84.5	83.4
0-10		7.6	8.2
>10-20		6.2	6.6
>20		1.7	1.8
Suspicion of parametrial invasion	392 (17.5%)		
Absence		92.6	92.1
Presumably absent		1.7	1.8
Presumably present		5.7	6.1
Tumour size, cm	49 (2.2)		
≤2		31.0	30.7
<4		8.2	8.2
>2-4		33.4	33.3
>4		27.4	27.9

Numbers represent % or number of patients. [a] The short-axis diameter of suspicious common iliac nodes was included in the model but not imputed, as it was only missing for four patients.

Abbreviations: cN, clinical nodal status; LVSI, lympho-vascular space invasion.

Supplementary Table 4. Patient-based diagnostic indices for MRI, CT and [^{18}F]FDG-PET-CT in detecting lymph node metastases of patient cohorts with multiple imaging results.

Cohort	n	Modality	AUC of original data[a]	n	AUC of imputed data[a]
MRI + [^{18}F]FDG-PET-CT	114	MRI	0.706 (0.615-0.798)	314	0.749 (0.684-0.815)
		[^{18}F]FDG-PET-CT	0.792 (0.710-0.873)		0.814 (0.746-0.882)
MRI + CT	234	MRI	0.647 (0.580-0.713)	384	0.713 (0.656-0.771)
		CT	0.613 (0.548-0.678)		0.655 (0.599-0.710)
[^{18}F]FDG-PET-CT + CT	40	[^{18}F]FDG-PET-CT	0.773 (0.634-0.914)	106	0.796 (0.694-0.898)
		CT	0.685 (0.523-0.847)		0.722 (0.614-0.829
MRI + CT + [^{18}F]FDG-PET-CT	23	MRI	0.631 (0.410-0.851)	59	0.703 (0.559-0.847)
		CT	0.673 (0.458-0.888)		0.668 (0.522-0.814)
		[^{18}F]FDG-PET-CT	0.769 (0.575-0.964)		0.762 (0.614-0.911)

[a] AUC (95% confidence interval) without dichotomising the nodal status on imaging.

Abbreviations: n, number of patients.

Supplementary Table 5. The prevalence of lymph node metastases in patient cohorts according to (multiple) imaging results.

Cohorts	n	MRI	CT	[^{18}F]FDG-PET-CT	Prev LNM (%)
	1,390	Negative			15
	286	Positive			66
	778		Negative		18
	148		Positive		64
	196			Negative	18
	183			Positive	76
1	283	Negative	Negative		17
2	20	Negative	Positive		38
3	31	Positive	Negative		65
4	50	Positive	Positive		74
5	40		Negative	Negative	15
6	16		Negative	Positive	73
7	11		Positive	Negative	52
8	39		Positive	Positive	78
9	126	Negative		Negative	18
10	31	Negative		Positive	58
11	21	Positive		Negative	14
12	136	Positive		Positive	79
13	17	Negative	Negative	Negative	19
14	22	Positive	Positive	Positive	71

Based on imputed data for cohorts with n>10.

Abbreviations: n, number of patients; Prev LNM, prevalence of lymph node metastases.

Supplementary Table 6. Patient-based diagnostic indices for MRI, CT and [^{18}F]FDG-PET-CT in detecting lymph node metastases based on original and imputed data, inconclusive nodes considered negative.

Modality	Prev LNM	Sensitivity	Specificity	PPV	NPV	AUC[a]
Original data						
MRI	19 (17-22)	24 (21-26)	98 (97-98)	70 (67-72)	84 (82-86)	0.605 (0.577-0.633)
CT	24 (21-28)	27 (24-31)	97 (96-98)	75 (71-79)	81 (77-84)	0.623 (0.585-0.660)
[^{18}F]FDG-PET-CT	44 (35-52)	68 (61-76)	88 (83-94)	82 (76-88)	78 (71-85)	0.783 (0.714-0.853)
Imputed data						
MRI	24 (22-26)	38 (35-40)	96 (95-97)	77 (75-79)	83 (81-85)	0.671 (0.642-0.700)
CT	26 (23-29)	31 (28-33)	97 (96-98)	77 (74-79)	80 (78-83)	0.636 (0.600-0.672)
[^{18}F]FDG-PET-CT	46 (41-51)	75 (71-79)	85 (81-89)	81 (77-85)	80 (76-84)	0.795 (0.740-0.850)

Numbers represent % with (95% confidence interval). [a] AUC with dichotomising the nodal status on imaging.

Abbreviations: Prev LNM, prevalence of lymph node metastases.

Supplementary Table 7. Region-based diagnostic indices for MRI, CT and [^{18}F]FDG-PET-CT in detecting lymph node metastases based on original and imputed data, inconclusive nodes considered negative.

Modality	Region	Prev LNM	Sensitivity	Specificity	PPV	NPV	AUC[a]
Original data							
MRI	Pelvic	19 (16-21)	23 (21-25)	97 (97-98)	67 (64-68)	85 (83-87)	0.602 (0.574-0.630)
	Common iliac	4 (3-5)	8 (6-9)	100 (-)	100 (-)	96 (95-98)	0.539 (0.496-0.581)
CT	Pelvic	23 (20-27)	25 (22-29)	97 (96-98)	72 (69-76)	81 (78-84)	0.612 (0.574-0.650)
	Common iliac	4 (2-6)	0 (-)	100 (99-100)	0 (-)	96 (94-98)	0.499 (0.497-0.501)
[^{18}F]FDG-PET-CT	Pelvic	42 (34-51)	68 (60-76)	89 (84-95)	83 (76-89)	79 (72-86)	0.787 (0.716-0.857)
	Common iliac	16 (9-23)	13 (6-19)	99 (97-100)	67 (58-76)	86 (79-93)	0.557 (0.472-0.641)
Imputed data							
MRI	Pelvic	23 (21-25)	38 (36-41)	96 (95-97)	75 (73-77)	84 (82-86)	0.673 (0.645-0.700)
	Common iliac	8 (7-9)	9 (8-10)	100 (99-100)	71 (70-73)	93 (91-94)	0.540 (0.512-0.578)
CT	Pelvic	25 (22-28)	29 (26-32)	97 (96-98)	76 (73-79)	80 (78-83)	0.629 (0.593-0.665)
	Common iliac	8 (7-10)	2 (1-3)	100 (99-100)	34 (31-37)	92 (90-93)	0.509 (0.487-0.543)
[^{18}F]FDG-PET-CT	Pelvic	45 (40-50)	74 (69-78)	85 (82-89)	80 (76-84)	80 (76-84)	0.795 (0.720-0.870)
	Common iliac	22 (18-26)	17 (13-21)	96 (94-98)	53 (49-58)	80 (76-84)	0.566 (0.490-0.642)

Numbers represent % with (95% confidence interval). [a] AUC with dichotomising the nodal status on imaging.

Abbreviations: Prev LNM, prevalence of lymph node metastases.

Supplementary Figure S1. Patient-flow chart of patient-based analyses according to Standards for Reporting of Diagnostic Accuracy (STARD).

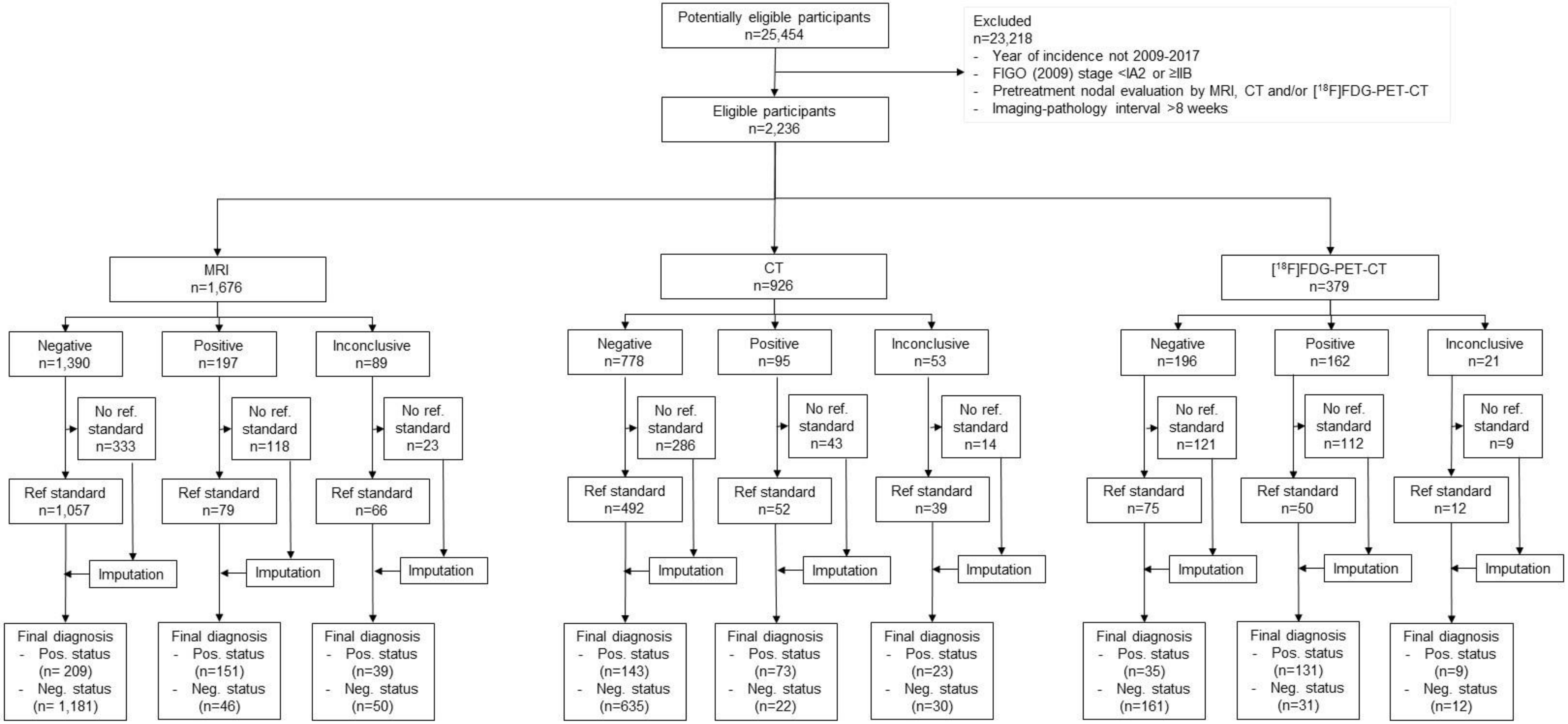

Supplement: Supplementary file 1 — Additional file 1: Supplementary Table 1. Imputation models. Supplementary Table 2. Distribution of variables with missing data before and after multiple imputation on patient-based level. Supplementary Table 3. Distribution of variables with missing data before and after multiple imputation on region-based level. Supplementary Table 4. Patient-based diagnostic indices for MRI, CT and [18F]FDG-PET-CT in detecting lymph node metastases of patient cohorts with multiple imaging results. Supplementary Table 5. The prevalence of lymph node metastases in patient cohorts according to (multiple) imaging results. Supplementary Table 6. Patient-based diagnostic indices for MRI, CT and [18F]FDG-PET-CT in detecting lymph node metastases based on original and imputed data, inconclusive nodes considered negative. Supplementary Table 7. Region-based diagnostic indices for MRI, CT and [18F]FDG-PET-CT in detecting lymph node metastases based on original and imputed data, inconclusive nodes considered negative. Supplementary Figure S1. Patient-flow chart of patient-based analyses according to Standards for Reporting of Diagnostic Accuracy (STARD). [file 13244_2023_1589_MOESM1_ESM.pdf]
